# Generation of Fibrin-Based Aortic Vessels with Layer-Specific Cell Architecture Under Pulsatile Perfusion in a Clinical Organ Care System

**DOI:** 10.1007/s10439-026-04087-9

**Published:** 2026-03-27

**Authors:** Celina Delia Käding, Clara-Sophie Glomb, Patrick Stadler, Imke Becker, Melanie Klingenberg, Hans-Klaus Höffler, Michael Pflaum, Arjang Ruhparwar, Mathias Wilhelmi, Florian Helms

**Affiliations:** 1 Lower Saxony Center for Biomedical Engineering, Implant Research and Development (NIFE), Hannover Medical School, Hannover, Germany; 2 Division for Cardiothoracic-, Transplantation-, and Vascular Surgery, Hannover Medical School, Hannover, Germany; 3 Department of Vascular- and Endovascular Surgery, St. Bernward Hospital, Hildesheim, Germany

**Keywords:** Vascular graft, Bioreactor, Pulsatile perfusion, Tissue engineering, Aortic prostheses

## Abstract

**Purpose:**

Overcoming the limitations of synthetic vascular grafts in the development of biocompatible and regenerative vessels remains a long-term objective in tissue engineering. In this study, we engineered large-diameter fibrin-based vascular grafts with a target inner diameter of 21 mm replicating all three layers of the native human vessel wall in vitro.

**Methods:**

The *Tunica media* was reconstructed using a compressed high-density (25 mg/mL) fibrin matrix seeded with smooth muscle cells expressing α-SMA and calponin, differentiated from adipose-derived mesenchymal stem cells (ASCs). The *Adventitia*-equivalent was formed in a 5 mg/mL fibrin gel containing ASCs, human umbilical vein endothelial cells (HUVECs), and normal human dermal fibroblasts (NHDFs) to enable the formation of a capillary-like network resembling the native adventitial *Vasa vasorum*. The luminal surface was endothelialized with HUVECs to replicate the *Tunica intima*. While controls were cultured statically for 7 days, other grafts were evaluated under pulsatile perfusion at physiological pressures using the TransMedics^®^ “Organ Care System Heart^™^”.

**Results:**

The stepwise fabrication technique resulted in three-layered bioartificial vessel equivalents with a mean inner diameter of 21 mm. All constructed vessels (*n* = 3) maintained sufficient biomechanical stability to withstand physiological pressure (120.9 ± 1.2 to 61.4 ± 12.5 mmHg) at 60 bpm throughout the perfusion period. The aortic grafts exhibited a cyclic stretch (8.95 ± 2.03%) within the physiological range of native vessels. Mechanical stimulation induced a layer-specific physiological cell morphology and alignment across all three layers of the vascular wall. Endothelial coverage was high on static grafts (81.39 ± 2.37%) and on one of three dynamic grafts. Shear stress during perfusion (0.38 ± 0.03 dyn cm^−2^) remained below physiological levels, and further evaluation under prolonged perfusion (> 21 days) is required to assess long-term biomechanical stability and vascular functionality.

**Conclusion:**

This approach may be considered a proof of concept and constitutes a first step toward the development of biometric and functional large-diameter vessel replacements.

**Supplementary Information:**

The online version contains supplementary material available at https://doi.org/10.1007/s10439-026-04087-9.

## Introduction

With cardiovascular diseases remaining the leading cause of death worldwide [1], the demand for effective therapeutic strategies in this field continues to grow. Synthetic polymer-based vascular grafts are commonly used to replace large-diameter vessels, with polyethylene terephthalate (Dacron^®^) and polytetrafluoroethylene (PTFE) as the predominant prosthetic materials [2]. As non-biological and non-biodegradable implants [3], these grafts fail to promote cellular infiltration and tissue regeneration [4, 5]. This limitation increases the risk of biofilm formation and aortic graft infection, which represents one of the most severe postoperative complications after synthetic aortic graft implantation with reported incidences ranging from 1% to 4% and a high associated mortality rate [6].

Although synthetic grafts provide mechanical stability and durability, they do not replicate the biomechanical elasticity of the native vessels. This issue is particularly critical in aortic replacements, as up to 60% of total arterial compliance originates from the aorta [7]. The resulting compliance mismatch can induce systolic hypertension and contribute to cardiac hypertrophy in patients with pre-existing cardiovascular conditions [8].

Tissue engineered vascular grafts (TEVGs) represent a promising alternative to overcome these limitations of synthetic vascular graft materials by offering biocompatible, potentially autologous vessel replacements that fully integrate, grow, and adapt to the recipient’s body and replicate the physiological elastic properties of native vessels [9]. However, to generate biometric and functional vascular implants, it is essential to accurately replicate the physiological multi-layered architecture of the native vessel wall, which still today remains a major challenge for large-scale tissue engineering approaches:

The native blood vessel wall consists of three distinct layers, each characterized by specific cell types and physiological functions. The *Tunica intima* contains endothelial cells that form an antithrombogenic surface and facilitate a laminar blood flow. The *Tunica media* consists of contractile smooth muscle cells, which provide mechanical stability and regulate vascular tone. The *Tunica adventitia* includes fibroblasts and a microvascular network known as *Vasa vasorum* that facilitates nutrient and gas exchange. This adventitial vascular network might be particularly crucial in large-diameter vascular implants to enable proper metabolic and functional integration and nutrition of the bioartificial vessel wall as well as long-term graft patency and performance [10].

The successful reconstruction of this complex architecture in TEVGs requires the integration of multiple cell types. These cells must be embedded within a scaffold that supports their physiological morphology and functional behavior, ultimately enabling a gradual replacement of the original scaffold material [11]. For this purpose, our approach is based on a fibrin-based matrix. Fibrin, a key protein in hemostasis and wound healing, is widely used as a naturally derived biomaterial due to its excellent suitability to support cell adhesion, proliferation, and remodeling [12–15]. Its verified biocompatibility and biodegradability have led to its well-established clinical application as fibrin glue in various surgical fields with clinically proven hemo- and biocompatibility [16].

We have previously generated cellularized, fibrin-based small-diameter TEVGs [17, 18]. Other groups have developed multi-layered large-diameter grafts using polycarbonate polyurethane (PC-PU) or electrospun polyurethane [19, 20]. However, to date, no in vitro study has successfully replicated all three layers of the human vessel wall in large-diameter TEVGs. Consequently, the goal of providing bioactive and biomimetic vascular grafts, particularly for large-diameter and aortic vessel replacements, is yet unmet by the currently available regenerative approaches.

For the development and maturation of tissue-engineered constructs, bioreactors represent essential instruments. They mimic the tissue-specific biochemical and biomechanical environment and apply controlled stimuli to promote cellular orientation and proliferation, as well as enhance mechanical properties through extracellular matrix production (ECM) [21, 22]. Additionally, bioreactors enable ex vivo assessment of TEVGs under nearly physiological conditions. However, widely accessible, reliable, and especially clinically validated perfusion systems remain scarce.

In this study, we generated fibrin-based vascular grafts of aortic dimensions that replicate the three-layered vessel wall structure of the human aorta including layer-specific cell types. Furthermore, we exposed the grafts to in vitro pulsatile perfusion to assess their biomechanical performance and support their cellular maturation. For this, we modified the commercially available TransMedics^®^ Organ Care System [23] for ex vivo perfusion. We further evaluated the system’s ability to provide a physiological perfusion environment and the essential biomechanical stimuli for tissue maturation, potentially offering a more standardized and translatable approach for in vitro perfusion of large-scale tissue-engineered vascular grafts.

## Materials and Methods

### Fibrinogen Isolation and Cell Culture

Fibrinogen was cryoprecipitated from human plasma obtained from the Blood Bank at Hannover Medical School (Institute for Transfusion Medicine) after written consent following a previously described protocol [24]. Adipose-derived mesenchymal stem cells (ASCs) were isolated from subcutaneous fat tissue of a patient undergoing visceral surgery at our institution after informed consent and ethical approval. ASC isolation, culture, and biochemical pre-differentiation into smooth muscle cells (SMCs) were performed as previously described [25]. Pre-differentiation was evaluated morphologically via light microscopy and confirmed by immunostaining for alpha-smooth muscle actin (α-SMA) as an early-stage marker, and calponin as an intermediate-stage marker of differentiation into a myogenic phenotype (Supplementary Fig. 1). Commercially available red-fluorescent protein-expressing human umbilical vein endothelial cells (HUVECs, Pelo Biotech, Planegg/Martinsried, Germany) were cultured in endothelial growth medium 2 (EGM2, Lonza, Basel, Switzerland) and used between passages 4 and 8. Normal human dermal fibroblasts (NHDFs, PromoCell GmbH, Heidelberg, Germany) were cultured in Dulbecco’s Modified Eagle’s Medium (DMEM, Thermo Fisher Scientific, Waltham, MA, USA) with 10% fetal bovine serum (FBS, Pan Biotech, Aidenbach, Germany).

### Generation of Three-layered Aortic Vascular Grafts

Cellularized three-layered vessels were generated using a stepwise fabrication process, beginning with the *Tunica media*, followed by the *Tunica adventitia* and the *Tunica intima*.

**Fig. 1:**
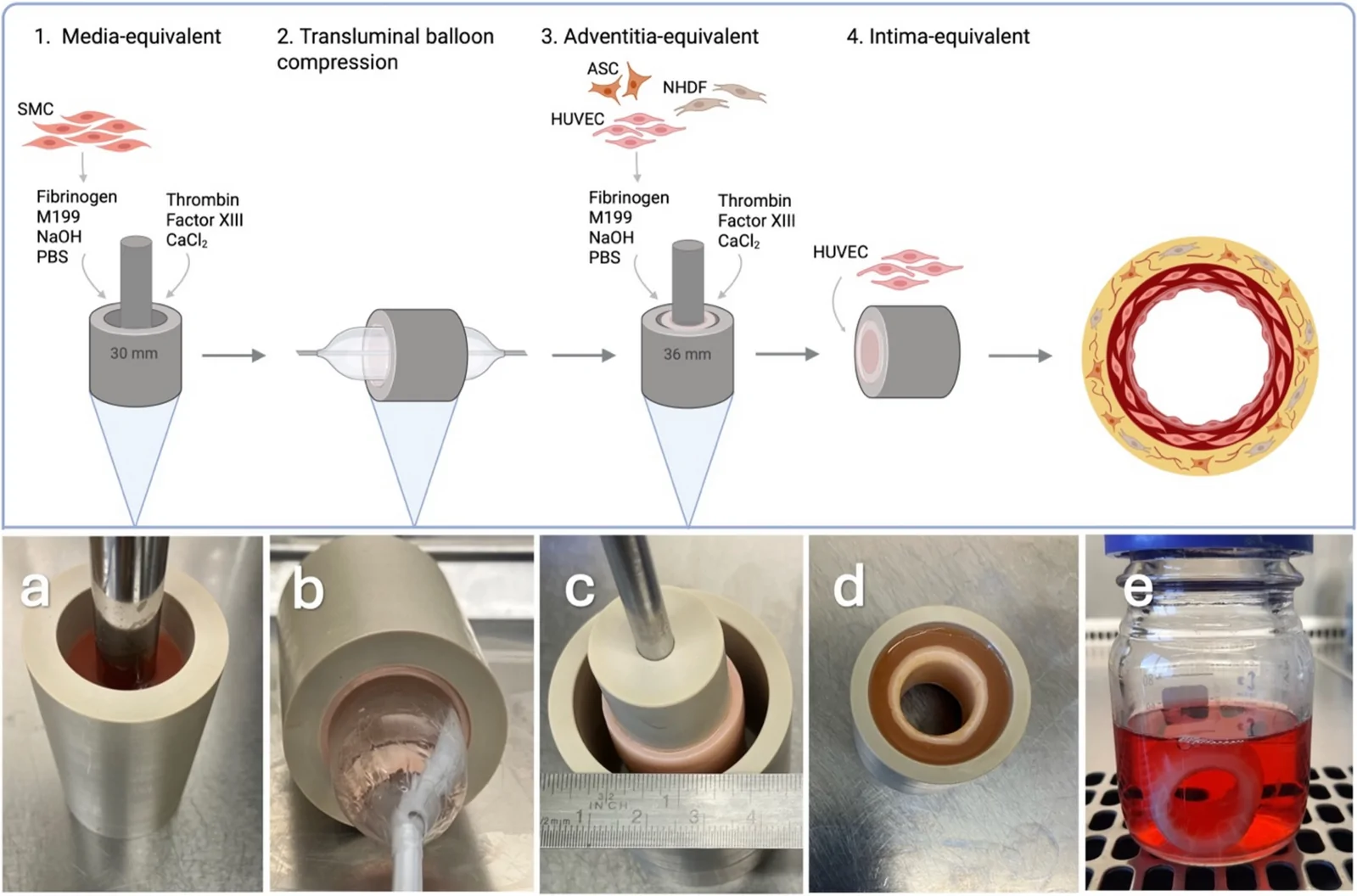
Construction of three-layered aortic vascular grafts in consecutive steps: **a** The fibrinogen solution containing SMCs is combined with the thrombin solution within a 3 cm molding form. **b** After static polymerization, transluminal balloon compression of the vessel wall was conducted. **c** Subsequently, the Adventitia-equivalent containing HUVECs, ASCs, and NHDFs was molded around the Tunica media-equivalent. **d** Endothelial cells for the generation of the Intima-equivalent were added inside the vessel lumen. The vessel was incubated either statically (**e**) or dynamically in feeding medium

For the construction of the *Media*-equivalent, 2.5 × 10^6^ SMCs/ml were suspended in a solution containing 25 mg/mL fibrinogen, 100 μL/mL 10 × M199 Medium (Sigma Aldrich, St. Louis, MO, USA), 100 U/mL Aprotinin (Loxo GmbH, Dossenheim, Germany) to prevent fibrin degradation and 1 N NaOH for pH adjustment. A second solution was prepared, containing 2.5 U/mL thrombin, 2.5 U/mL factor XIII (CSL Behring, Marburg, Germany) and 40 mM CaCl_2_. Both components with a total volume of 30 mL were mixed in a custom-built mold (Fig. 1a) with a 30 mm inner diameter and 15 mm central placeholder (Department of Medical Device Construction, Hannover Medical School). After 15 min of static polymerization, the placeholder was replaced with an achalasia balloon with a 30 mm diameter and 80 mm length (Endo-Flex, Voerde, Germany, Fig. 1b). The balloon expansion resulted in compression of the vessel wall. Pressure was set to 1 bar using an inflation device (Basix Compak, Merit Medical, South Jordan, UT, USA) and maintained at 1 bar for 30 min. Subsequently, the compressed *Media*-equivalent was placed around a 21 mm placeholder and inside a 36 mm inner diameter molding form (Fig. 1c).

To fabricate the *Adventitia*-equivalent, 1 × 10^6^/mL RFP-HUVECs, 2.5 × 10^5^/mL NHDFs and 2.5 × 10^5^/mL ASCs were suspended in a solution containing 5 mg/mL fibrinogen. The two solutions were then combined as described above within the 36 mm mold to initiate polymerization. After 30 min of static incubation, the central placeholder was removed (Fig. 1d).

In the final step, the *Intima-*equivalent was constructed by incubating 2.5 × 10^5^/mL HUVECs suspended in EGM2 containing 100 U/mL Aprotinin within the vessel lumen for 60 min. For this, the vessel was placed horizontally and rotated around the vessel axis for 90° every 15 min. Subsequently, the fibrin-based three-layered aortic graft was cultured in feeding medium containing M199 (Sigma Aldrich), 40 ng/mL hFGF, 40 ng/mL VEGF (both from PeproTech, Cranbury, NJ, USA) 50 μg/mL l-ascorbic acid-2-phosphate (Sigma Aldrich), Penicillin/Streptomycin (AppliChem, Darmstadt, Germany), and 100 U/mL Aprotinin (Loxo GmbH, Dossenheim, Germany) under either static conditions (Fig. 1e) or under dynamic perfusion in the OCS for 7 days.

For orientational biomechanical characterization, 10 mm vessel segments were prepared, and uniaxial tensile strength and elastic modulus were measured immediately after vessel fabrication, as previously described [26].

### Pulsatile Perfusion within the OCS^™^

**Fig. 2:**
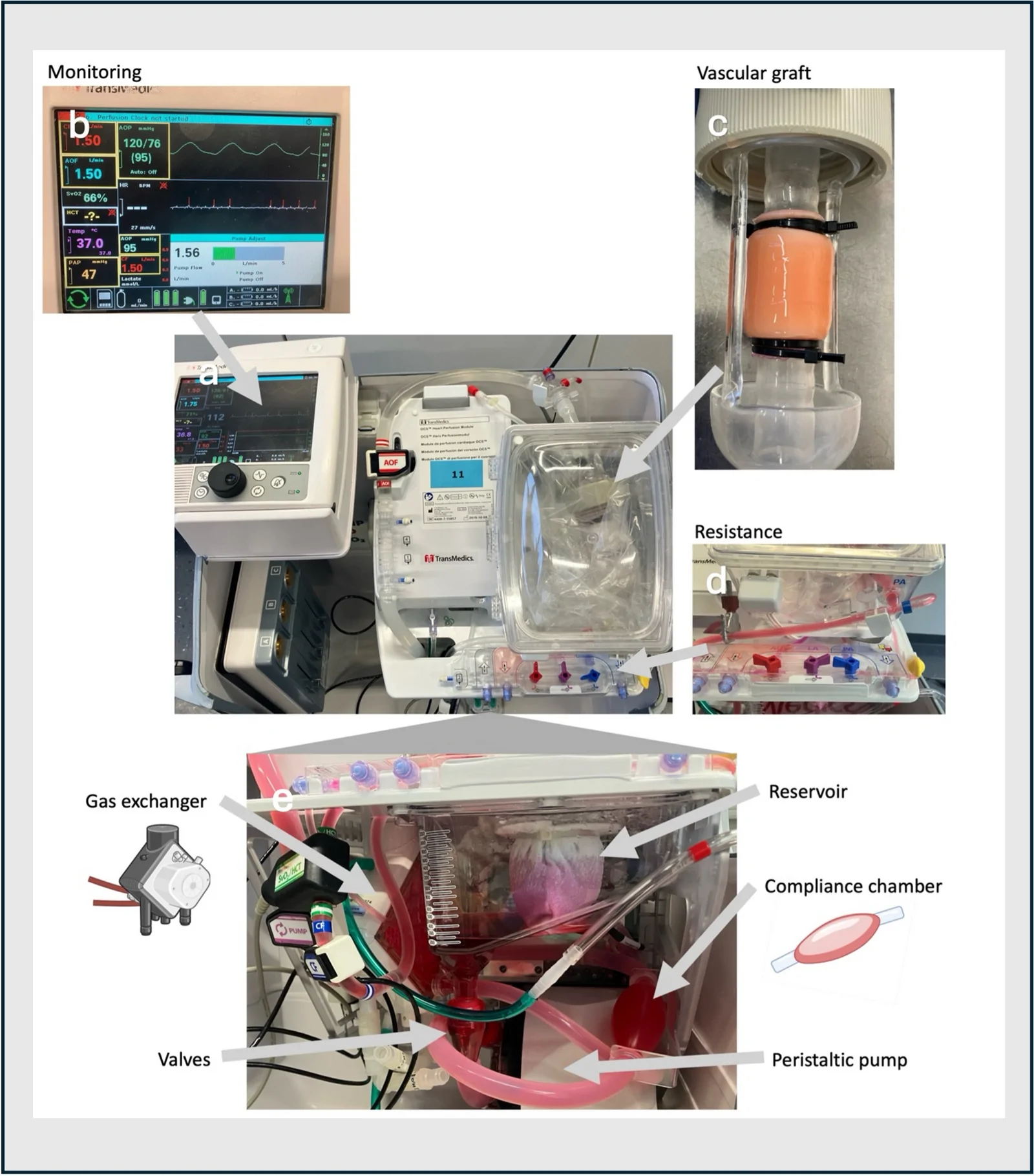
Overview of the TransMedics^®^ Organ Care System Heart (**a**): A monitor displayed the circulation parameters including pulse frequency, systolic and diastolic pressure, flow, and temperature (**b**). The vascular graft was placed inside the custom-built bioreactor (**c**), which was connected via tubing in the heart perfusion chamber of the OCS. Flow was adjusted using the pump control and pressure was set by varying the resistance of the backflow into the reservoir (**d**). Pulsatile flow was produced by a discontinuous pump with two valves (**e**). A compliance chamber was used to adjust the pressure amplitude and to generate positive diastolic pressure. External gas supply was facilitated through a gas exchanger

The TransMedics^®^ Organ Care System Heart (OCS^™^, TransMedics, Andover, USA) is a clinically tested and approved circulation system designed for ex vivo perfusion of donor hearts during transplantations. In this study, the system was modified for dynamic perfusion of the generated aortic grafts. The entire insert was gas sterilized prior to use. Fig. 2 provides an overview of the components of the perfusion system: Flow was generated by a peristaltic pump. Its discontinuous pulse was enhanced by two valves, positioned before and after the pump module. The compliance chamber installed downstream of the pump reduced the pressure drop between pulses and thus increased diastolic pressure to adjust the pulse amplitude. CO_2_ from an external gas blender was supplied through an oxygenator. Temperature was controlled with a heat-exchange unit. Pulse frequency, flow, pressure, and temperature were continuously recorded by integrated sensors of the OCS (pressure sensor sampling rate: 50 Hz) and displayed on a monitor (Fig. 2b). No zeroing or calibration of the sensors was performed, as sensor calibration is conducted during manufacturing and integrated into the OCS system. Flow was adapted via the pump control panel. Pressure was modified using the resistance board and an additional clamp (Fig. 2d).

For pulsatile perfusion, the graft was connected to adapters with a 20 mm diameter within a custom-built bioreactor (Fig. 2c, Department of Medical Device Construction). Priming was conducted with feeding medium. The bioreactor was then installed inside the OC system in the heart perfusion chamber. After an initial 48 h of static incubation to allow cell adhesion, the flow was increased gradually within 6 h to the targeted parameters shown below. Perfusion was initiated at 0.49 ± 0.02 L/min and subsequently increased every 1 to 2 h to 0.65 ± 0.0 L/min, 0.74 ± 0.01 L/min, 0.99 ± 0.01 L/min and 1.50 ± 0.01 L/min. Pulse frequency, flow, pressure, and temperature were monitored and recorded continuously during the perfusion period. Gas concentrations and pH in the circulating medium were measured once per day and adjusted manually by blood gas analysis (ABL800 FLEX analyzer, Radiometer Medical ApS, Copenhagen, Denmark).

To compare perfusion conditions within the OCS to physiological parameters and to estimate the impact of pulsatile flow on HUVECs, shear stress was calculated using the Poiseuille equation. The following simplifying assumptions were applied: laminar and steady flow, a Newtonian and homogeneous fluid, and a cylindrical vessel geometry.

Shear Stress (*τ*) was calculated as follows (Eq. 1):

1 $$\tau = \frac{4\mu Q}{\pi r3},$$

where *μ* represents the viscosity of the medium, *Q* is the volume flow, and *r* is the vessel radius.

The inner radius was measured on unfixed vessels immediately after explanation and represents an approximation of mean vessel radius under perfusion conditions. The viscosity of the medium was assumed to be 1.0 mPa s, and a volumetric flow rate of 1.5 L/min was used.

Cyclic stretch of the vascular graft was quantified by measuring the outer vessel diameter at the central region of the graft at systolic and diastolic pressure in video recordings, as described previously [17]. As changes in diameter translate proportionally into changes in circumference, cyclic stretch was calculated assuming a circular cross-section using the following equation (Eq. 2):

$$\text{Cyclic\; stretch} \left(\%\right)=\left(\frac{D_{\mathrm{syst}}}{{D_{\mathrm{dia}}}}-1\right)\times 100,$$

where *D*_syst_ represents the vessel diameter at systolic pressure and *D*_dia_ is the diameter at diastolic pressure.

Additionally, vascular graft compliance was calculated as follows (Eq. 3):

3 $$\mathrm{Distensibility}\;\left(\mathrm{mmHg}^{-1}\right)= \frac{\left(\frac{\left(D_{\mathrm{syst}} - D_{\mathrm{dia}}\right)}{{D}}\right)}{{P_{\mathrm{syst}}}- {P_{\mathrm{dia}}}},$$

where *D* represents the vessel diameter, *P*_syst_ represents the systolic pressure, and *P*_dia_ represents the diastolic pressure.

### Analysis of Cellularized Three-Layered Grafts

Three vascular grafts per group were generated as described above and analyzed after 7 days of either static or pulsatile incubation.

For the evaluation of the *Media*-equivalent, vessels were analyzed for cell distribution and viability as well as smooth muscle cell marker expression and cell morphology and alignment. In the first step, live-dead staining was performed by washing the vessel twice in PBS followed by incubation with calcein and ethidium homodimer (viability/cytotoxicity kit, Life Technologies, Darmstadt, Germany) for 45 min, followed by visualization using fluorescent microscopy (Nikon Eclipse TE300, Düsseldorf, Germany). Cell distribution and viability were assessed throughout the vessel wall and live-dead cells were quantified in 5 fields in 10× magnification using ImageJ (NIH, Bethesda, MD, USA). After fixation in 4% paraformaldehyde for at least 24 h, 10 µm cryosections were prepared. Slides were stained for smooth muscle cell specific markers. Therefore, sections were permeabilized with 0.1% Triton-X-100, blocked with PBS + 10% FCS, 0.1% BSA + 0.05% Tween-20 (all from Sigma Aldrich) and incubated with TRITC-labeled phalloidin (Sigma Aldrich) or primary antibodies: Mouse anti-alpha-smooth muscle actin (1:100, DM001, Acris Antibodies, Herford, Germany) and Rabbit anti-calponin (1:100; ab46794; Abcam, Cambridge, UK). As secondary antibodies, Alexa Fluor 488-coupled anti-rabbit IgG and Alexa Fluor 555-coupled anti-mouse IgG (Thermo Fisher Scientific, Waltham, MA, USA) were used. Nuclei were stained with 4,6-diamidino-2-phenylindole (DAPI, Carl Roth GmbH, Karlsruhe, Germany).

The *Adventitia*-equivalent was assessed by fluorescence microscopy by placing the outer surface of 10 mm long vessel segments on a microscopic slide. Capillary tube formation was quantified in 6 fields per vessel in 4× magnification using the Software AngioTool (Version 0.6; setting for all images: minimum intensity: 15; maximum intensity: 255; threshold: 7) [27]. To visualize the tube network throughout the vessel wall and verify capillary formations in all layers of the *Adventitia*, optical clearing was performed by incubating the vascular grafts in a 5:3 mixture of methyl salicylate and benzyl benzoate (MSBB, both from Sigma Aldrich) after dehydration in an ascending ethanol series. Images were captured using confocal laser scanning microscopy (Leica TCS SP5, Leica Microsystems, Wetzlar, Germany).

To visualize endothelial cell coverage of the *Intima*-equivalent, 10 mm long segments were cut longitudinally. The luminal layer of the graft was analyzed in 6 fields per vessel in 10× magnification. Endothelial coverage was quantified using the software ImageJ^®^. Endothelial alignment was quantified using OrientationJ (ImageJ), with the longitudinal vessel axis defined as 0°.

### Statistics

Statistics were performed using SPSS (Version 29, Armonk, NY, USA). Normality was tested using the Shapiro–Wilk normality test, and homoscedasticity was evaluated using Levene’s test. Comparisons between two groups were performed using the Mann–Whitney U test or unpaired Student’s *t* test. The *p* values were adjusted for multiple comparisons using the Benjamini–Hochberg method. The results were considered significant at *p* < 0.05. No power estimations were conducted for this proof-of-concept study due to the limited number of vessels per group (*n* = 3 per group). Therefore, all results should be regarded as exploratory.

## Results

### Perfusion Conditions in the OCS^™^

Perfusion of the vascular grafts in the OCS provided sterile incubation conditions throughout the perfusion period. The system facilitated continuous monitoring of flow, pressure, and temperature. The perfusion parameters remained stable during the perfusion period. Measured values and the corresponding physiological values of the native human aorta are shown in Table 1.

**Table 1:** Perfusion parameters during dynamic incubation in comparison with physiological conditions in the native human aorta [28–34]

	Measured	Physiological
Pulse frequency (bpm)	60	60–90 [31]
Flow (L/min)	1.5 ± 0.01	5–6 [32]
Systolic pressure (mmHg)	120.9 ± 1.2	90–120 [30]
Diastolic pressure (mmHg)	61.4 ± 12.5	60–80 [30]
Temperature (°C)	37.0	36.1–37.2 [33]
Shear Stress (dyn cm^−2^)	0.38 ± 0.03	10–70 [29]
Cyclic stretch (%)	8.95 ± 2.03	5–10 [28]
Distensibility (×10^−3^ mmHg^−1^)	1.50 ± 0.32	1.86 ± 1.31 [34]

Values are given as mean ± standard deviation

**Fig. 3:**
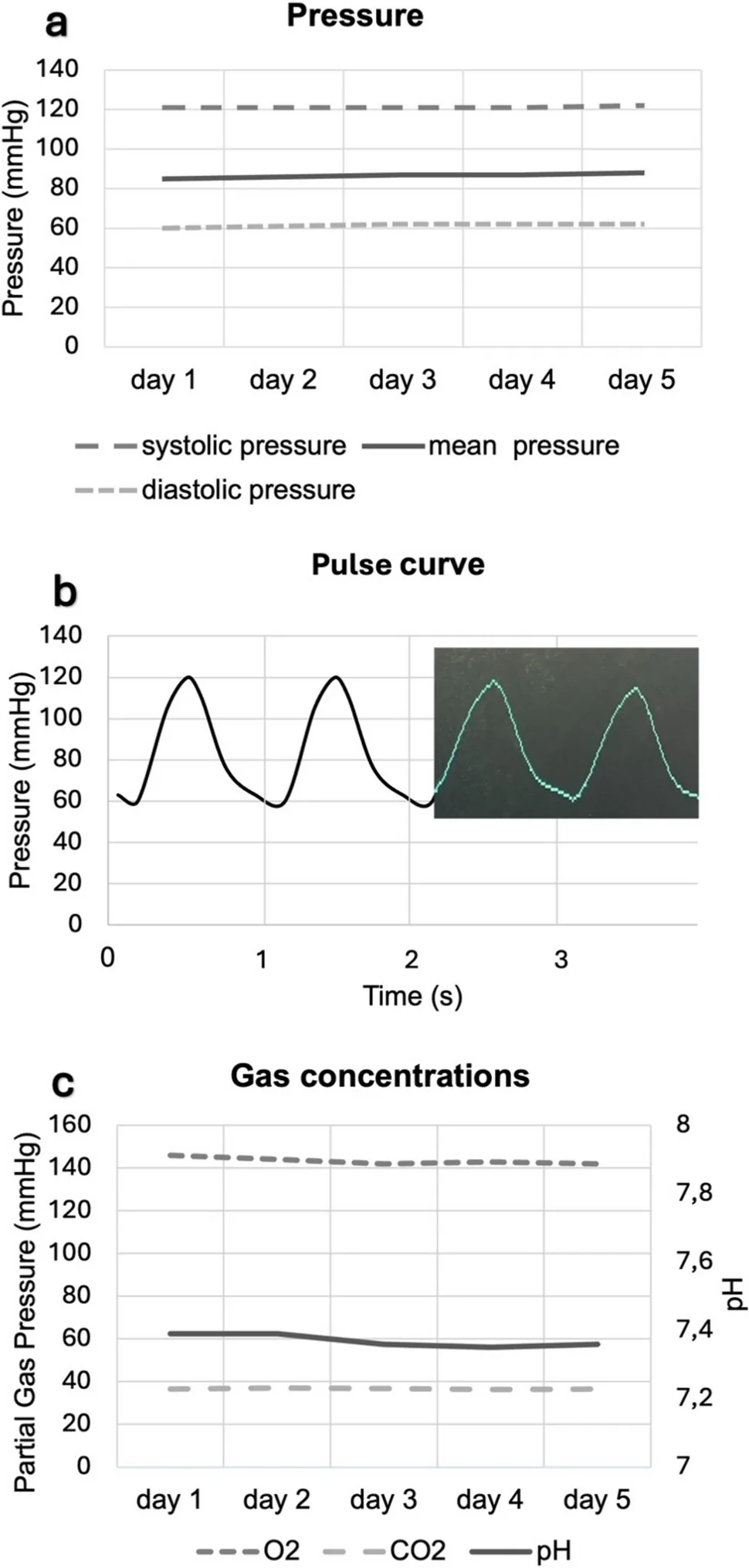
Perfusion conditions were documented continuously in the OCS and through daily blood gas analysis. **a** Systolic, diastolic, and mean pressure progression over the incubation period. **b** Exemplary illustration of the pulse pressure curve at mean systolic and diastolic pressure; the inset depicts the original reading from the OCS monitor. **c** pH, O_2_, and CO_2_ concentrations during the dynamic incubation period

Figure 3 illustrates stable pressure values throughout the incubation period (Fig. 3a), with fluctuations of less than 4% in mean systolic (121 to 122 mmHg), mean (85 to 88 mmHg), and diastolic pressure (60 to 62 mmHg) between perfusion days. Exemplary raw pressure and flow recordings are provided as supplementary material (Supplementary Table 1). Gas concentrations and pH remained stable throughout the perfusion period (Fig. 3c), with mean O_2_ partial pressure between 142 and 146 mmHg, CO_2_ between 36.3 and 37.0 mmHg, and pH between 7.35 and 7.39, corresponding to variations of less than 3% between perfusion days. Pulse frequency, pressure, temperature, pH (7.37 ± 0.02), and gas concentrations (CO_2_ = 36.65 ± 0.22 mmHg, O_2_ = 143.4 ± 1.5 mmHg) fell within the physiological circulatory ranges (Table 1). In contrast, flow and shear stress were lower than in the human circulatory system. The pulse curve exhibited a waveform resembling the physiological aortic pressure curve (Fig. 3b). No relevant pressure, gas, or flow fluctuations (< 5%) were observed during the perfusion period (Table 1, Fig. 3a, c).

### Macroscopic Characterization of the Vascular Grafts

A total of six vascular grafts with aortic dimensions were fabricated, each with an initial inner diameter of approximately 21 mm, a *Media* thickness of 2 mm, an *Adventitia* thickness of 4 mm, and a total length of 40 mm. Measurements after 7 days of incubation are presented in comparison with physiological abdominal and thoracic aortic dimensions of the native human aorta in Table 2. Both statically and dynamically cultured grafts exhibited a reduction in inner diameter and wall thickness. This reduction was significantly more evident in static vessels (79.87 ± 3.87% of the original inner diameter) compared to dynamically perfused grafts (90.0 ± 2.52%, unpaired *t*-Test, adjusted *p* = 0.048, mean difference: 10.13, 95% CI 2.74–17.53; Cohen’s *d* = 3.11).

**Table 2:** Dimensions of aortic vascular grafts after 7 days of either static or dynamic maturation compared to physiological parameters [31–34]

	Static incubation	Dynamic incubation	adjusted* p* value	Natural thoracic aorta	Natural abdominal aorta
Luminal diameter (mm)	16.77 ± 0.81	18.90 ± 0.53	*0.048 (mean difference: 2.13; 95% CI (0.57–3.68); Cohen*’*s d* = *3.11)*	29–35 [36]	15–27 [37]
*Media* thickness (mm)	1.93 ± 0.11	1.84 ± 0.16	0.444 (mean difference: − 0.10; 95% CI (− 0.41 to 0.22); Cohen’s *d* = − 0.69)	1.63 ± 0.36 [35]	1.49 ± 0.75 [38]
*Adventitia* thickness (mm)	3.44 ± 0.51	2.57 ± 0.32	0.112 (mean difference: − 0.87; 95% CI (− 1.84 to 0.10); Cohen’s *d* = − 2.04)		
Outer diameter (mm)	22.14 ± 0.99	23.30 ± 0.51	0.125 (U = 0, Median_stat_ = 22.70, Median_dyn_ = 23.13, Cliff’s delta = − 1)		

Values are given as mean ± standard deviation. Unpaired *t* test or Mann–Whitney *U* test for Outer diameter

All constructed vessels maintained sufficient biomechanical stability to withstand physiological pressure throughout the perfusion period. Tensile testing showed an ultimate tensile strength of 178 ± 32 kPa and a secant elastic modulus of 110 ± 28 kPa. We observed a cyclic stretch of 8.95 ± 2.03% at the stated pressure pulse (120.9 ± 1.2 to 61.4 ± 12.47 mmHg) and a distensibility of 1.50 ± 0.32 × 10^−3^ mmHg^−1^.

### Assessment of the Three-Layered Vessel Wall and Cell Morphology After Static or Dynamic Incubation

#### Tunica Media

**Fig. 4:**
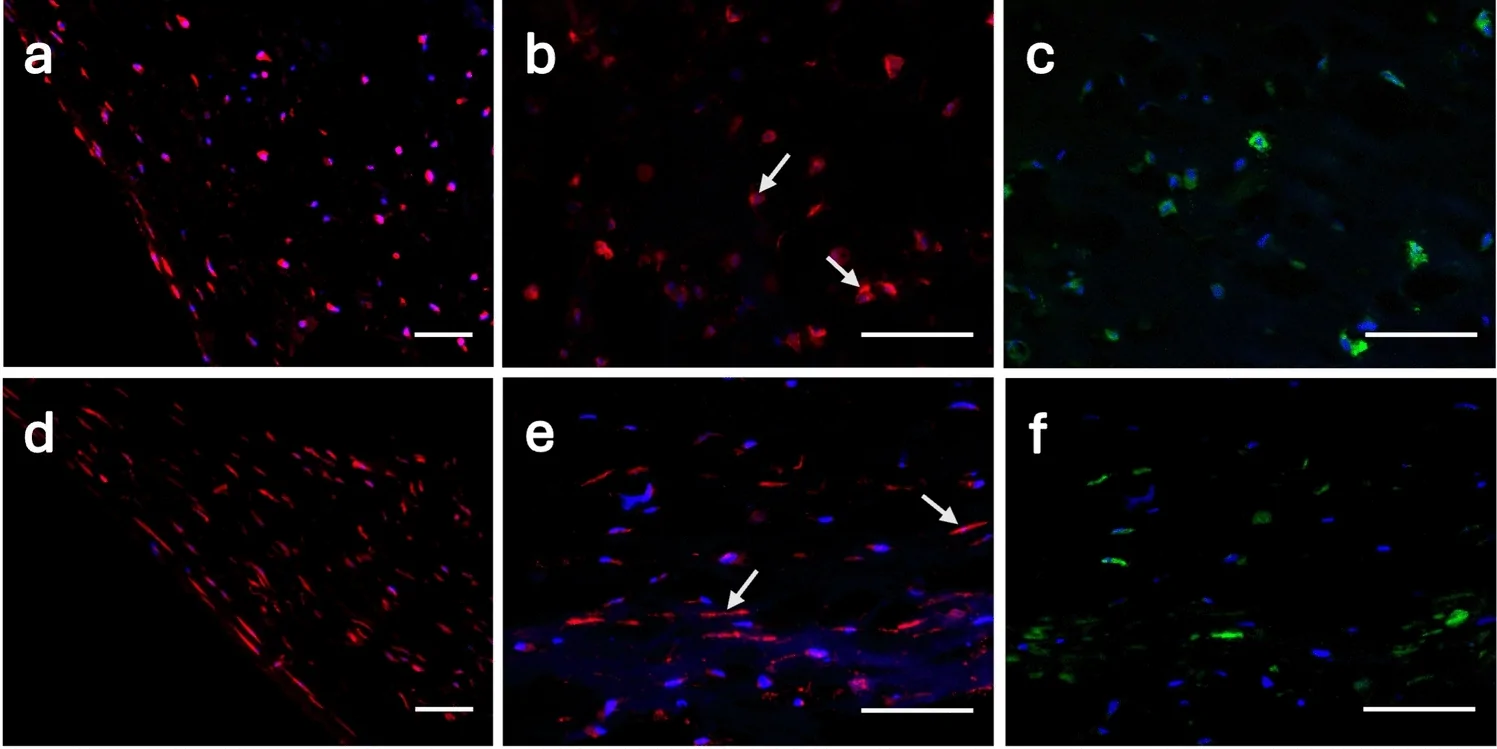
Circumferential cryosections of the Tunica media-equivalent after static (**a–c**) or dynamic incubation (**d–f**). Nuclei staining was performed with DAPI (blue), slides were stained for phalloidin (red, **a**, **d**), α-SMA (red, **b**, **e**), or Calponin (green, **c**, **f**). Scale bar = 100 µm

Evaluation of the *Media*-equivalent revealed a homogeneous distribution and viability of SMCs throughout the vessel wall (Supplementary Fig. 2). Live/dead staining indicated higher cell viability (96.6 ± 2.6%) in static grafts, compared to dynamically perfused grafts (81.6 ± 8.4%, Mann–Whitney *U*-test, *p* = 0.100, Median_stat_ = 97.5, Median_dyn_ = 86.2, Cliff’s delta = 1). Immunostaining for α-SMA and calponin visualized SMCs in the *Media* and verified the maintenance of a myogenic phenotype in both groups during the incubation period (Fig. 4). Undifferentiated ASCs and NHDFs in the *Adventitia* did not exhibit the expression of α-SMA and calponin (Supplementary Fig. 3). Furthermore, SMCs in the *Media* of mechanically stimulated grafts appeared in an organized arrangement: In statically incubated grafts, SMCs exhibited a round, non-aligned morphology, whereas in dynamically perfused vessels, SMCs demonstrated an elongated phenotype with circumferential alignment around the vessel lumen (exemplary arrows in Fig. 4).

### Tunica Adventitia

**Fig. 5:**
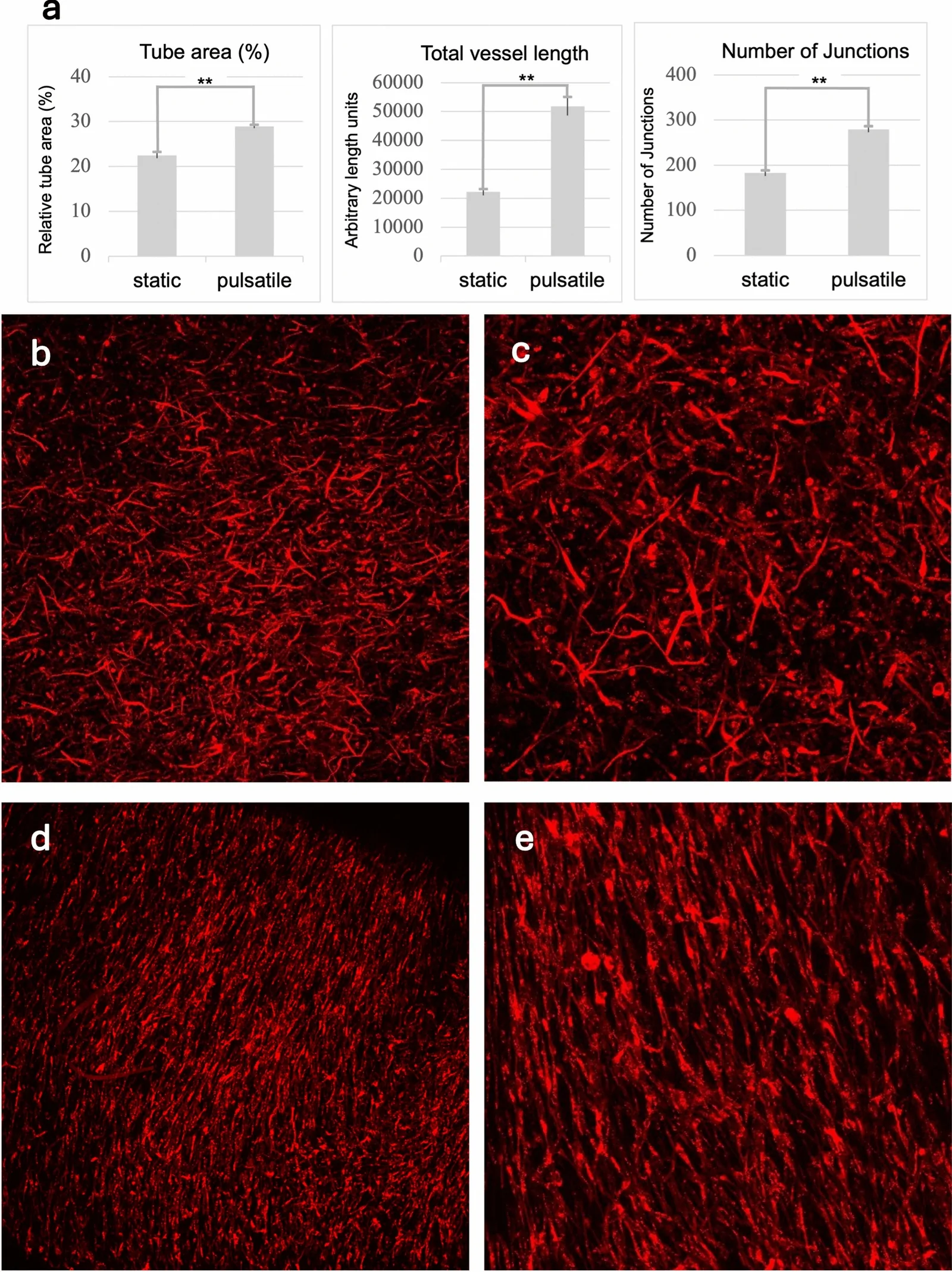
Assessment of the Adventitia-equivalent: visualization of the capillary network throughout the vessel wall after optical clearing by confocal laser microscopy. Z-stack images were created of 500 µm of the Adventitia-equivalent of static (**b**, **c**) and dynamic (**d**, **e**) vascular grafts in 2.9 µm steps. Quantitative analysis of tube area, total vessel length, and number of junctions of capillary tubes was performed using AngioTool (**a**). Values are given as mean ± SEM. ×10 magnification (**b**, **d**) and ×20 magnification (**c**, **e**)

The combination of HUVECs, ASCs, and NHDFs in a 4:1:1 ratio embedded in a 5 mg/mL fibrin matrix enabled the formation of capillary-like structures by HUVECs (Fig. 5b–e). The addition of dynamic perfusion resulted in a significantly larger area covered by capillary tube formations as analyzed using the AngioTool^®^ Software (Fig. 5a, Supplementary Fig. 4, unpaired *t*-test, adjusted *p* = 0.003, 22.49 ± 1.16 vs 28.89 ± 0.69, mean difference: 6.40, 95% CI 4.23–8.47, Cohen’s *d* = 6.68). Moreover, capillary length and number of junctions increased after mechanical stimulation compared to static controls (unpaired *t*-test, corrected *p* = 0.003, Junctions: 182.72 ± 9.81 vs 280.17 ± 12.58, mean difference: 97.45, 95% CI 71.88–123.02, Cohen’s *d* = 8.64, vessel length: corrected *p* = 0.003, 22257.67 ± 1721.15 vs 51823.67 ± 5492.32, mean difference: 29566.0, 95% CI 20399.74–38792.26, Cohen’s *d* = 7.27). Additionally, a pronounced longitudinal alignment of these formations along the vessel axis could be observed. Confocal laser microscopy visualized the capillary network in the vessel wall and indicated the presence of tube formations throughout the wall (Fig. 5).

### Tunica Intima

**Fig. 6:**
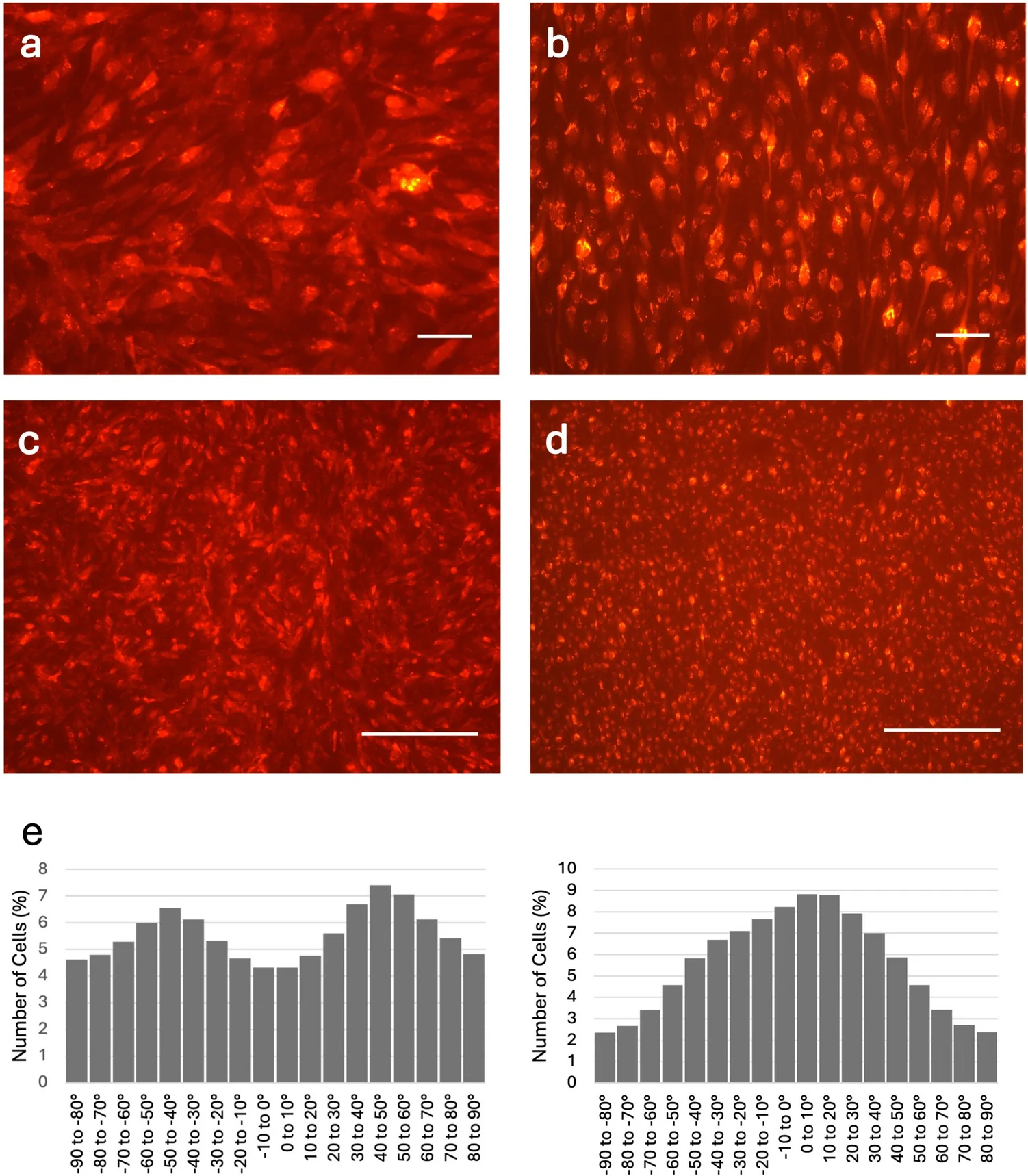
Evaluation of the Intima-equivalent: fluorescence microscopy of the luminal surface of static (**a**, **c**) and dynamic (**b**, **d**) vessels. RFP-HUVECs are visualized in red. Quantification of endothelial orientation on static (left) and dynamic grafts (right, **e**). Scale bar **a**, **b** = 100 µm, **c**, **d** = 500 µm

Statically incubated vascular grafts exhibited a confluent layer of endothelial cells. In contrast to HUVECs in the *Adventitia*, no tube formation was observed in the *Intima*. Among the three vessels which were exposed to dynamic perfusion within the OCS, only one showed a confluent monolayer of RFP-HUVECs (Fig. 6). Quantification of the endothelial confluency in ImageJ revealed a high coverage (81.39 ± 2.37%) for static grafts and in the single confluent dynamic graft (80.63 ± 1.88%). These values were comparable to the baseline endothelial confluence determined after 12 h of static incubation in preliminary experiments (82.19 ± 2.11%). However, HUVECs under OCS perfusion appeared elongated and aligned with the direction of flow (Fig. 6b, d). Quantitative analysis revealed a unimodal distribution centered around the vessel axis (0°) in dynamically incubated grafts (Fig. 6e). In contrast, HUVECs on static grafts exhibited an orientated cellular arrangement that was not aligned with the vessel axis.

## Discussion

In this study, we generated cellularized, fibrin-based vascular grafts with aortic dimensions. In summary, the key outcomes are as follows: (1) The stepwise fabrication process enabled the generation of three-layered grafts with aortic dimensions. (2) The bioartificial fibrin grafts replicated each layer of the vessel wall with their specific cell types. (3) Mechanical stimulation enhanced physiological cell alignment in all three layers and capillary formation in the *Adventitia*-equivalent. (4) The OCS provided stable physiological perfusion conditions throughout the perfusion period and represents a promising system for assessment and maturation of tissue-engineered constructs.

To the best of our knowledge, this proof-of-concept study represents the first in vitro realization of a fibrin-based, three-layered vessel of aortic dimensions. Fibrin is a well-studied, biocompatible, and biodegradable material that can be autologously derived from human plasma [39, 40]. In previous rheological evaluations, fibrin has been characterized as a compliant, gel-like material with low storage modulus [41]. A major limitation of fibrin is its insufficient mechanical strength [40]. To address this, we upscaled a previously established balloon compression technique (Fig. 1) to enhance mechanical stability by increasing fibrin matrix density, as recently demonstrated using electron microscopy [42]. The comparable *Media* wall thickness across all vessels indicated a reproducible wall density achieved by balloon compression (Table 2). This yielded vascular grafts with sufficient biomechanical properties to withstand physiological pressures during short-term ex vivo perfusion. Nevertheless, further studies extending the perfusion duration of these initial proof-of-concept experiments are required to assess long-term stability. Future biomechanical characterization should extend the orientational mechanical testing conducted in this study and include additional assessments such as burst pressure, suture holding strength, as well as residual stress and viscoelastic properties, to enable a more accurate comparison with physiological blood vessels. Previous biomechanical testing of fibrin-based small-diameter grafts and cardiac patches indicated the need for further improvements in mechanical stability, potentially including reinforcements with spider silk and other materials to ensure reliable graft performance under supraphysiological perfusion parameters [42–44]. Replicating native elasticity in TEVGs is critical to prevent systolic hypertension and cardiac remodeling [8, 45]. In contrast to currently used synthetic prostheses, which show no elastic and compliant properties, our generated grafts exhibited a cyclic stretch of 8.95 ± 2.03%, which lies within the physiological range of native vessels (5–10%) [28]. Graft distensibility (1.50 ± 0.32 × 10^−3^ mmHg^−1^) was comparable to physiological measurements reported in adults without cardiovascular disease aged 45 to 84 years (1.86 ± 1.31 × 10^−3^ mmHg^−1^) [34]. Thus, the bioartificial aortic vessels closely approximated the physiological compliance behavior of native vessels.

### Emulation of the Three-Layered Vessel Wall

The replication of the complex architecture of native blood vessels, with distinct functional roles assigned to each layer, is essential to generate biologically active and biomimetic vascular implants. One of the closest in vitro realizations of this objective in larger-diameter vessels was achieved by Hoerstrup *et al*., who fabricated pre-seeded TEVGs for pulmonary artery replacement with an outer diameter of 18± 1 mm, using a polyglycolic acid (PGA) mesh coated with poly-4-hydroxybutyrate (P_4_HB) [46]. Although these grafts demonstrated adequate mechanical strength, they exhibited reduced elasticity compared to native vessels. As the degradation of PGA scaffolds can induce inflammatory responses [47], our grafts were constructed from a biocompatible fibrin matrix and with slightly larger diameters and higher compliance suitable for aortic replacement (Table 2). Moreover, while the cellularization of the PGA-P_4_HB grafts was limited to two layers, one seeded with endothelial cells and the other with myofibroblasts, thus lacking the adventitial layer, which is of pivotal importance for graft integration and vessel wall nutrition, the fibrin-based grafts generated in this study were composed of all three layers of the native vessel wall in vitro.

### Tunica Media

For the replication of the *Tunica media,* SMCs were differentiated from ASCs, which are autologously accessible and highly multipotent [48]. The fibrinogen concentration of 25 mg/mL, combined with balloon compression, resulted in a stable matrix within the physiological range of native aortic wall thickness (Table 2). The homogeneous distribution and viability of SMCs throughout the *Medi*a indicated no relevant cell death during the fabrication process including balloon compression. After 7 days of incubation, the expression of α-SMA and calponin confirmed a contractile SMC phenotype in both groups [46] (Fig. 4). The circumferential alignment of SMCs around the vessel lumen, induced by mechanical stimulation, is consistent with previous observations [17, 18] and resembles the physiological organization of vascular SMCs. Cyclic stretch has been identified as a key stimulus for SMC alignment, and the underlying cellular mechanisms have been described previously [49]. Thus, the cyclic stretch generated in the OCS facilitated a physiological circular SMC alignment in the aortic fibrin grafts as well. Circular SMC alignment represents a crucial requirement for proper functionality. We have previously demonstrated contractile responses of SMCs embedded in fibrin-based small-diameter grafts [17]. Further evaluations after prolonged perfusion durations (> 21 days) are necessary to evaluate the inducible contractility of fibrin-based aortic grafts.

### Tunica Adventitia

Reconstructing the microvasculature of the *Tunica adventitia* in pre-vascularized tissues is pivotal to overcome the ~ 200 µm limit for passive gas and nutrient diffusion and to support a successful integration into the recipient’s microvasculature [50, 51]. ECM synthesis by fibroblasts in the *Adventitia* essentially determines the biomechanical properties of the vessel wall. In addition, as the *Tunica adventitia* is involved in inflammatory responses and vascular remodeling [10], its successful replication may enhance immune compatibility, remodeling capacity, and long-term graft patency.

Here, we reconstructed the *Tunica adventitia* in fibrin by using a combination of three cell types to replicate the densely capillarized morphology of the native *Tunica adventitia*: A 5 mg/mL fibrin matrix was selected to imitate the extracellular structure of the *Adventitia*, while also supporting capillary tube formation [52]. Umbilical vein endothelial cells were chosen for their accessibility, previous phenotypic characterization, including platelet endothelial cell adhesion molecule (CD31), endothelial nitric oxide synthase (eNOS), von Willebrand factor (vWF), and vascular endothelial cadherin (VE-cadherin) expression, and their proven ability to form lumenized capillary-like structures in fibrin [18, 24, 53, 54]. Moreover, we have previously demonstrated that ASCs in HUVEC-Co-Culture express pericyte-like markers, including neural/glial antigen 2 (NG2) and platelet-derived growth factor receptor beta (PDGFRβ) and enhance capillary development in fibrin gels [54, 55]. Other groups studied the effect of fibroblasts on endothelial sprouting in different scaffold materials: for example, Velazquez et al. demonstrated an improvement in capillary formation by combining endothelial cells with dermal fibroblasts in collagen [56]. In this study, both approaches were combined by incorporating the three different cell types (HUVECs, ASCs, NHDFs) to accurately emulate the *adventitial* complexity. With NHDFs, we included a cell type specialized for ECM production. However, ECM synthesis and remodeling require extended culture durations, ranging from several weeks to months [13, 57]. Accordingly, extensive ECM deposition is not expected after the comparatively short perfusion period of 7 days in this study. For instance, Syedain et al. observed significant collagen remodeling and burst pressures up to 1400–1600 mmHg in 4 mg/mL fibrin-based grafts with embedded fibroblasts after 7–9 weeks of perfusion. However, as the vessel lacked initial stability, 2 weeks of static pre-maturation was necessary before the graft could be exposed to perfusion [58]. In this study, the reinforced fibrin matrix of the *Media*-equivalent compensated for the lower mechanical strength of the fibrin matrix used for the *Adventitia*, providing sufficient biomechanical stability for immediate perfusion. Moreover, the combination of the three different cell types, HUVECs, ASCs and NHDFs resulted in dense capillary network formation within the *Adventitia* in both groups (Fig. 5). We here observed an improvement in tube density, capillary length, and number of junctions within the capillary network under cyclic pulsatile perfusion. This contrasts with our previous studies, in which dynamic grafts exhibited fewer junctions than static grafts and comparable tube density [18]. Besides the difference in cell combinations (ASC + HUVEC vs. ASC + NHDF + HUVEC), this might be due to the increased perfusion duration in this study (3 days vs 7 days). While capillary networks in static grafts have previously exhibited structural degradation after 4 days of static incubation [59], this effect does not apply to capillary structures under biomechanical stimulation [41]. The longitudinal alignment of capillary tubes (Fig. 5) was likely induced by the combination of cyclic stretch and longitudinal tension applied during perfusion, as recently demonstrated in small-diameter grafts [60].

### Tunica Intima

A functional endothelium with antithrombogenic properties is a major objective in the generation of TEVGs. Here, static incubation of vascular grafts supported the formation of a confluent endothelial monolayer with high coverage (81.39 ± 2.37%). However, two of three grafts under dynamic perfusion exhibited areas with no endothelial coverage or only residues of cells. This is most likely due to mechanical damage and air exposure during the installation and explantation process in the OCS. As the initial venting of the system during the start of perfusion requires a certain amount of flow, the sudden increase might also affect endothelial survival and attachment. Nevertheless, one perfused graft did not exhibit areas without cell coverage but a high confluency (80.63 ± 1.88%). As this was the final experiment, the improved outcome may be attributed to more experienced handling, particularly in minimizing air bubbles during system venting. Furthermore, no additional coatings were applied in this study. Previous studies have shown that coatings such as heparin or other surface modifications can enhance endothelial attachment [61], representing a promising strategy for future experiments. In contrast to randomly oriented HUVECs on static grafts, endothelial cells on dynamically perfused grafts aligned in the direction of flow [Fig. 6 b, d]. Endothelial alignment in response to laminar flow requires sufficient mechanosensory transduction of shear stress, stable cell–cell, and cell–matrix interaction and reorganization of the cellular cytoskeleton [62]. Despite the substantially lower application of shear stress (0.38 ± 0.03 dyn cm^−2^) compared to physiological calculations (10–70 dyn cm^−2^) [28], the parallel alignment of HUVECs suggested cellular maintenance of the above-described functions on large-diameter fibrin vessels during the 7-day perfusion period. Quantitative analysis confirmed a preferential endothelial alignment around the vessel axis (0°) (Fig. 6 e) but did not show the pronounced orientation observed under physiological shear stress in previous studies [18]. Furthermore, prolonged exposure to reduced shear stress affects endothelial morphology and function by modulating gene expression, cytoskeletal arrangement, cellular permeability, and vascular reactivity, ultimately promoting atherogenesis, inflammation and vessel wall remodeling [63, 64]. Thus, prospective studies could increase shear stress by using dextran or by replacing the circulating medium with blood, additionally enabling an evaluation of pre-endothelialization on the hemocompatibility of fibrin grafts. As no specific endothelial marker characterization was performed in this proof-of-concept study, prospective studies are necessary to further assess endothelial phenotypical expression and functional properties, including nitric oxide release and thrombogenicity.

### Pre-cellularized Vascular Implants and Potential for In Vivo Remodeling

Pre-cellularization prior to implantation has been shown to enhance graft integration and function. However, the cost and time required for fabricating such constructs, especially when using autologous cell sources, may limit clinical application. Compared to other, more time-consuming bioengineering approaches [39, 58, 65, 66], our vessels were generated within a relatively short period of 7 days. Still, the necessity of complete pre-cellularization and the potential for in vivo cellularization and maturation remain to be investigated. Previous in vivo studies of fibrin-based pre-cellularized vessels have demonstrated the infiltration of calponin-positive SMCs and capillaries from surrounding tissue after 6 months of implantation [26]. Row *et al.* compared hybrid fibrin scaffolds pre-seeded with both ECs and SMCs versus ECs alone. Interestingly, while SMC-pre-seeding improved cellular ingrowth and host remodeling, it was not an essential requirement. Both groups exhibited a comparable contractile function after explantation [67]. These findings suggest that partial pre-seeding might be a compromise to achieve functional, well-integrated implants. However, the short duration of these initial proof-of-concept experiments limits the evaluation of three-layered vascular grafts for long-term application. Future in vivo studies in large animal models are necessary to assess long-term remodeling, degradation behavior, and immune compatibility within the physiological environment.

### The OCS^™^ as a Bioreactor for Tissue Engineering

Several studies have shown that pulsatile perfusion provides the essential mechanical stimuli for maturation of engineered tissues into functional, biomimetic constructs [68, 69]. In this study, the OCS, originally used for donor heart preservation during transplantation, was used for the perfusion of vascular grafts. To our knowledge, this represents the first application of this system in a vascular tissue engineering context. Unlike other customized bioreactors, which often require the assembly of multiple components to mimic the human circulation system, the OCS offers a standardized, commercially available framework for ex vivo perfusion. It is already authorized for medical application and provides sterile incubation conditions. Its integrated monitoring system facilitates a continuous documentation of relevant perfusion parameters. Biochemical conditions including pH, pO_2_, pCO_2_ (Fig. 3), and temperature remained stable during the whole perfusion period. Biomechanical stimuli resulted in physiological systolic and diastolic pressures (120.9 ± 1.2 to 61.4 ± 12.47 mmHg) at a flow of 1.5 L/min. This flow was selected in accordance with perfusion rates typically applied for transplant hearts (0.9–1.2 L/min) [70] and remained lower than physiological flow rates in the human aorta. Furthermore, limited accessibility and associated costs of the system in comparison with custom-built bioreactors may restrict its widespread application. Nevertheless, the system successfully facilitated the assessment of biomechanical behavior including stability and compliance of the bioartificial grafts under physiological pressure conditions. Furthermore, it provided the biomechanical stimuli to promote capillary formation in the *Adventitia* and initiated physiological cellular alignment in all three layers of the fabricated grafts.

This work can be considered a first proof of concept for large-diameter bioengineered blood vessels from fibrin under dynamic perfusion. We demonstrated successful in vitro culture of different vessel-specific cell types under static and physiological conditions and observed physiological cellular reorganization. As a result, a fibrin-based bioartificial vessel with aortic dimensions and physiological cell morphology and alignment in all three layers of the vessel wall was constructed. Further research is needed concerning long-term mechanical stability, in vivo degradation and remodeling behavior as well as vascular functionality and overall influence of cellularization on graft performance.

## Supplementary Information

Below is the link to the electronic supplementary material.

Supplementary file1 (DOCX 5362 KB)

## Data Availability

Further data used for the manuscript are available from the corresponding author upon reasonable request.

## References

[1] GBD 2021 Causes of Death Collaborators. Global burden of 288 causes of death and life expectancy decomposition in 204 countries and territories and 811 subnational locations, 1990–2021: a systematic analysis for the Global Burden of Disease Study 2021. *Lancet*. 403:2100–2132, 2024. 10.1016/S0140-6736(24)00367-2. PMC11126520 38582094

[2] Kannan, R. Y., H. J. Salacinski, P. E. Butler, G. Hamilton, and A. M. Seifalian. Current status of prosthetic bypass grafts: a review. *J. Biomed. Mater. Res. B Appl. Biomater.* 74B(1):570–581, 2005. 10.1002/jbm.b.30247. 15889440

[3] Blumenberg, R. M., J. M. Anderson, M. L. Gelfand, P. S. Skudder, and C. A. Bowers. Histologic evaluation of Dacron and PTFE graft material explanted from humans after 4 to 20 years in vivo. *Vasc. Endovasc. Surg.* 34(6):505–512, 2000. 10.1177/153857440003400601.

[4] Müller, K. M., and G. Dasbach. The pathology of vascular grafts. *Curr. Top. Pathol.* 86:273–306, 1994. 10.1007/978-3-642-76846-0_7. 8162712

[5] Tsuchida, H., S. E. Wilson, and S. Ishimaru. Healing mechanisms of high-porosity PTFE grafts: significance of transmural structure. *J. Surg. Res.* 71:187–195, 1997. 10.1006/jsre.1997.5158. 9299289

[6] Berger, P., I. Vaartjes, F. L. Moll, G. J. De Borst, J. D. Blankensteijn, and M. L. Bots. Cumulative incidence of graft infection after primary prosthetic aortic reconstruction in the endovascular era. *Eur. J. Vasc. Endovasc. Surg.* 49:581–585, 2015. 10.1016/j.ejvs.2015.01.001. 25736515

[7] Stergiopulos, N., P. Segers, and N. Westerhof. Use of pulse pressure method for estimating total arterial compliance in vivo. *Am. J. Physiol. Heart Circ. Physiol.* 276:H424–H428, 1999. 10.1152/ajpheart.1999.276.2.H424. 9950841

[8] Spadaccio, C., F. Nappi, N. Al-Attar, F. W. Sutherland, C. Acar, A. Nenna, M. Trombetta, M. Chello, and A. Rainer. Old myths, new concerns: the long-term effects of ascending aorta replacement with dacron grafts. Not all that glitters is gold. *J. Cardiovasc. Transl. Res.* 9:334–42, 2016. 10.1007/s12265-016-9699-8. PMC4990605 27245785

[9] Pashneh-Tala, S., S. MacNeil, and F. Claeyssens. The tissue-engineered vascular graft-past, present, and future. *Tissue Eng. Part B Rev.* 22:68–100, 2016. 10.1089/ten.teb.2015.0100. PMC4753638 26447530

[10] Stenmark, K. R., M. E. Yeager, K. C. El Kasmi, E. Nozik-Grayck, E. V. Gerasimovskaya, M. Li, S. R. Riddle, and M. G. Frid. The adventitia: essential regulator of vascular wall structure and function. *Annu. Rev. Physiol.* 75:23–47, 2013. 10.1146/annurev-physiol-030212-183802. PMC3762248 23216413

[11] Iqbal, N., A. S. Khan, A. Asif, M. Yar, J. W. Haycock, and I. U. Rehman. Recent concepts in biodegradable polymers for tissue engineering paradigms: a critical review. *Int. Mater. Rev.* 64:91–126, 2019. 10.1080/09506608.2018.1460943.

[12] Ahmann, K. A., J. S. Weinbaum, S. L. Johnson, and R. T. Tranquillo. Fibrin degradation enhances vascular smooth muscle cell proliferation and matrix deposition in fibrin-based tissue constructs fabricated in vitro. *Tissue Eng. Part A* 16:3261–3270, 2010. 10.1089/ten.tea.2009.0708. PMC2947425 20536358

[13] Ross, J. J., and R. T. Tranquillo. ECM gene expression correlates with in vitro tissue growth and development in fibrin gel remodeled by neonatal smooth muscle cells. *Matrix Biol.* 22:477–490, 2003. 10.1016/s0945-053x(03)00078-7. 14667840

[14] Cummings, C. L., D. Gawlitta, R. M. Nerem, and J. P. Stegemann. Properties of engineered vascular constructs made from collagen, fibrin, and collagen-fibrin mixtures. *Biomaterials.* 25:3699–3706, 2004. 10.1016/j.biomaterials.2003.10.073. 15020145

[15] Vardoulis, O., E. Coppens, B. Martin, P. Reymond, P. Tozzi, and N. Stergiopulos. Impact of aortic grafts on arterial pressure: a computational fluid dynamics study. *Eur. J. Vasc. Endovasc. Surg.* 42:704–710, 2011. 10.1016/j.ejvs.2011.08.006. 21889370

[16] Rousou, J. A. Use of fibrin sealants in cardiovascular surgery: a systematic review. *J. Card Surg.* 28:238–247, 2013. 10.1111/jocs.12099. 23578196

[17] Helms, F., S. Lau, M. Klingenberg, T. Aper, A. Haverich, M. Wilhelmi, and U. Böer. Complete myogenic differentiation of adipogenic stem cells requires both biochemical and mechanical stimulation. *Ann Biomed Eng.* 48:913–926, 2020. 10.1007/s10439-019-02234-z. 30815762

[18] Helms, F., S. Lau, T. Aper, S. Zippusch, M. Klingenberg, A. Haverich, M. Wilhelmi, and U. Böer. A 3-layered bioartificial blood vessel with physiological wall architecture generated by mechanical stimulation. *Ann. Biomed. Eng.* 49:2066–2079, 2021. 10.1007/s10439-021-02728-9. 33483842

[19] Gu, Y., C. Tian, Y. Qin, Y. Sun, S. Liu, H. Li, X. Duan, C. Shu, and C. Ouyang. The novel hybrid polycarbonate polyurethane/polyester three-layered large-diameter artificial blood vessel. *J. Biomater. Appl.* 36:965–975, 2022. 10.1177/08853282211033415. 34284662

[20] Krammer, J., M. Pichlmaier, J. Stana, C. Hagl, S. Peterss, M. Grab, and L. Grefen. Multi-layered electrospun grafts for surgical repair: Biomimicking physiological ascending aortic compliance. *J. Appl. Biomater. Funct. Mater.* 23:22808000251316730, 2025. 10.1177/22808000251316728. 39921458

[21] Huang, A. H., and L. E. Niklason. Engineering of arteries in vitro. *Cell Mol. Life Sci.* 71:2103–2118, 2014. 10.1007/s00018-013-1546-3. PMC4024341 24399290

[22] Hansmann, J., F. Groeber, A. Kahlig, C. Kleinhans, and H. Walles. Bioreactors in tissue engineering - principles, applications and commercial constraints. *Biotechnol. J.* 8:298–307, 2013. 10.1002/biot.201200162. 23161827

[23] Pinnelas, R., and J. A. Kobashigawa. *Ex vivo* normothermic perfusion in heart transplantation: a review of the TransMedics Organ Care System. *Future Cardiol.* 18:5–15, 2022. 10.2217/fca-2021-0030. 34503344

[24] Lau, S., D. Eicke, M. Carvalho Oliveira, B. Wiegmann, C. Schrimpf, A. Haverich, R. Blasczyk, M. Wilhelmi, C. Figueiredo, and U. Böer. Low immunogenic endothelial cells maintain morphological and functional properties required for vascular tissue engineering. *Tissue Eng. Part A* 24:432–447, 2018. 10.1089/ten.TEA.2016.0541. 28978275

[25] Lau, S., M. Klingenberg, A. Mrugalla, F. Helms, D. Sedding, A. Haverich, M. Wilhelmi, and U. Böer. Biochemical myogenic differentiation of adipogenic stem cells is donor dependent and requires sound characterization. *Tissue Eng. Part A* 25:936–948, 2019. 10.1089/ten.TEA.2018.0172. 30648499

[26] Aper, T., M. Wilhelmi, C. Gebhardt, K. Hoeffler, N. Benecke, A. Hilfiker, and A. Haverich. Novel method for the generation of tissue-engineered vascular grafts based on a highly compacted fibrin matrix. *Acta Biomater.* 29:21–32, 2016. 10.1016/j.actbio.2015.10.012. 26472610

[27] Zudaire, E., L. Gambardella, C. Kurcz, and S. Vermeren. A computational tool for quantitative analysis of vascular networks. *PLoS One.*6:e27385, 2011. 10.1371/journal.pone.0027385. PMC3217985 22110636

[28] Jufri, N. F., A. Mohamedali, A. Avolio, and M. S. Baker. Mechanical stretch: physiological and pathological implications for human vascular endothelial cells. *Vasc. Cell.* 7:8, 2015. 10.1186/s13221-015-0033-z. PMC4575492 26388991

[29] Chatzizisis, Y. S., A. U. Coskun, M. Jonas, E. R. Edelman, C. L. Feldman, and P. H. Stone. Role of endothelial shear stress in the natural history of coronary atherosclerosis and vascular remodeling: molecular, cellular, and vascular behavior. *J. Am. Coll. Cardiol.* 49:2379–2393, 2007. 10.1016/j.jacc.2007.02.059. 17599600

[30] Schmieder, R. S., and H. Schunkert. Erhöhter Blutdruck und Bluthochdruck : ESC-Leitlinie 2024 rückt Risikoreduktion in den Fokus [Elevated blood pressure and hypertension: focus of the 2024 ESC guidelines on risk reduction]. *Herz.* 50:17–24, 2025. 10.1007/s00059-024-05285-8. 39589443

[31] Palatini, P. Need for a revision of the normal limits of resting heart rate. *Hypertension.* 33:622–625, 1999. 10.1161/01.hyp.33.2.622. 10024317

[32] King J, Lowery DR. Physiology, cardiac output. In: StatPearls [Internet]. Treasure Island: StatPearls Publishing, 2025. 29262215

[33] Yousef H, Ramezanpour Ahangar E, Varacallo MA. Physiology, thermal regulation. In: StatPearls [Internet]. Treasure Island: StatPearls Publishing; 2025 29763018

[34] Redheuil, A., C. O. Wu, N. Kachenoura, Y. Ohyama, R. T. Yan, A. G. Bertoni, G. W. Hundley, D. A. Duprez, D. R. Jacobs Jr., L. B. Daniels, C. Darwin, C. Sibley, D. A. Bluemke, and J. A. C. Lima. Proximal aortic distensibility is an independent predictor of all-cause mortality and incident CV events: the MESA study. *J. Am. Coll. Cardiol.* 64(24):2619–2629, 2014. 10.1016/j.jacc.2014.09.060. PMC4273646 25524341

[35] Shiran, H., J. Odegaard, G. Berry, D. C. Miller, M. Fischbein, and D. Liang. Aortic wall thickness: an independent risk factor for aortic dissection? *J. Heart Valve Dis.* 23:17–24, 2014. 24779324

[36] Turkbey, E. B., A. Jain, C. Johnson, A. Redheuil, A. E. Arai, A. S. Gomes, J. Carr, W. G. Hundley, G. Teixido-Tura, J. Eng, J. A. Lima, and D. A. Bluemke. Determinants and normal values of ascending aortic diameter by age, gender, and race/ethnicity in the Multi-Ethnic Study of Atherosclerosis (MESA). *J. Magn. Reson. Imaging* 39:360–368, 2014. 10.1002/jmri.24183. PMC3766427 23681649

[37] Mola, A. G., C. T. Díaz, G. G. Martins, X. T. Sari, and S. B. Montoya. Editor’s Choice - Systematic review and meta-analysis of normal infrarenal aortic diameter in the general worldwide population and changes in recent decades. *Eur. J. Vasc. Endovasc. Surg.* 64:4–14, 2022. 10.1016/j.ejvs.2022.04.014. 35483578

[38] Koc, A. S., and H. E. Sumbul. Increased aortic intima-media thickness may be used to detect macrovascular complications in adult type II diabetes mellitus patients. *Cardiovasc. Ultrasound.* 16(1):8, 2018. 10.1186/s12947-018-0127-x. PMC5996542 29891012

[39] Grassl, E. D., T. R. Oegema, and R. T. Tranquillo. A fibrin-based arterial media equivalent. *J. Biomed. Mater. Res. A* 66:550–561, 2003. 10.1002/jbm.a.10589. 12918038

[40] Shaikh, F. M., A. Callanan, E. G. Kavanagh, P. E. Burke, P. A. Grace, and T. M. McGloughlin. Fibrin: a natural biodegradable scaffold in vascular tissue engineering. *Cells Tissues Organs.* 188:333–346, 2008. 10.1159/000139772. 18552484

[41] Zippusch, S., F. Helms, S. Lau, M. Klingenberg, C. Schrimpf, A. Haverich, M. Wilhelmi, and U. Böer. Perfusion promotes endothelialized pore formation in high concentration fibrin gels otherwise unsuitable for tube development. *Int. J. Artif. Organs* 44:130–138, 2021. 10.1177/0391398820936700. 32611278

[42] Helms, F., A. Haverich, U. Böer, and M. Wilhelmi. Transluminal compression increases mechanical stability, stiffness and endothelialization capacity of fibrin-based bioartificial blood vessels. *J. Mech. Behav. Biomed. Mater.*124:104835, 2021. 10.1016/j.jmbbm.2021.104835. 34530301

[43] Glomb, C., M. Wilhelmi, S. Strauß, S. Zippusch, M. Klingenberg, T. Aper, P. M. Vogt, A. Ruhparwar, and F. Helms. Fabrication and biomechanical characterization of a spider silk reinforced fibrin-based vascular prosthesis. *J. Mech. Behav. Biomed. Mater.*152:106433, 2024. 10.1016/j.jmbbm.2024.106433. 38316085

[44] Bobylev, D., M. Wilhelmi, S. Lau, M. Klingenberg, M. Mlinaric, E. Petená, F. Helms, T. Hassel, A. Haverich, A. Horke, and U. Böer. Pressure-compacted and spider silk-reinforced fibrin demonstrates sufficient biomechanical stability as cardiac patch in vitro. *J. Biomater. Appl.* 36(6):1126–1136, 2022. 10.1177/08853282211046800. 34617818

[45] Reymond, P., N. Westerhof, and N. Stergiopulos. Systolic hypertension mechanisms: effect of global and local proximal aorta stiffening on pulse pressure. *Ann. Biomed. Eng.* 40:742–749, 2012. 10.1007/s10439-011-0443-x. 22016326

[46] Hoerstrup, S. P., I. Cummings Mrcs, M. Lachat, F. J. Schoen, R. Jenni, S. Leschka, S. Neuenschwander, D. Schmidt, A. Mol, C. Günter, M. Gössi, M. Genoni, and G. Zund. Functional growth in tissue-engineered living, vascular grafts: follow-up at 100 weeks in a large animal model. *Circulation.* 114:I159–I166, 2006. 10.1161/CIRCULATIONAHA.105.001172. 16820566

[47] Garimella, A., S. B. Ghosh, and S. Bandyopadhyay-Ghosh. Biomaterials for bone tissue engineering: achievements to date and future directions. *Biomed. Mater.* 2024. 10.1088/1748-605X/ad967c. 39577395

[48] Zhang, J., Y. Liu, Y. Chen, L. Yuan, H. Liu, J. Wang, Q. Liu, and Y. Zhang. Adipose-derived stem cells: current applications and future directions in the regeneration of multiple tissues. *Stem Cells Int.* 2020:8810813, 2020. 10.1155/2020/8810813. PMC7787857 33488736

[49] Rensen, S. S., P. A. Doevendans, and G. J. van Eys. Regulation and characteristics of vascular smooth muscle cell phenotypic diversity. *Neth. Heart J.* 15:100–108, 2007. 10.1007/BF03085963. PMC1847757 17612668

[50] Standley, P. R., A. Cammarata, B. P. Nolan, C. T. Purgason, and M. A. Stanley. Cyclic stretch induces vascular smooth muscle cell alignment via NO signaling. *Am. J. Physiol. Heart Circ. Physiol.* 283:H1907–H1914, 2002. 10.1152/ajpheart.01043.2001. 12384468

[51] Mazio, C., C. Casale, G. Imparato, F. Urciuolo, C. Attanasio, M. De Gregorio, F. Rescigno, and P. A. Netti. Pre-vascularized dermis model for fast and functional anastomosis with host vasculature. *Biomaterials.* 192:159–170, 2019. 10.1016/j.biomaterials.2018.11.018. 30453212

[52] Ben-Shaul, S., S. Landau, U. Merdler, and S. Levenberg. Mature vessel networks in engineered tissue promote graft–host anastomosis and prevent graft thrombosis. *Proc. Natl Acad. Sci. U.S.A.* 116:2955–2960, 2019. 10.1073/pnas.1814238116. PMC6386696 30718418

[53] Deng, D., Y. Zhang, B. Tang, and Z. Zhang. Sources and applications of endothelial seed cells: a review. *Stem Cell Res. Ther.* 15(1):175, 2024. 10.1186/s13287-024-03773-6. PMC11184868 38886767

[54] Lau, S., C. Schrimpf, M. Klingenberg, F. Helfritz, T. Aper, A. Haverich, M. Wilhelmi, and U. Böer. Evaluation of autologous tissue sources for the isolation of endothelial cells and adipose tissue-derived mesenchymal stem cells to pre-vascularize tissue-engineered vascular grafts. *BioNanoMaterials.* 16:309–321, 2015. 10.1515/bnm-2015-0014.

[55] Lau, S. Human adipose tissue-derived mesenchymal stem cells promote capillary formation of peripheral blood outgrowth endothelial cells in autologous fibrin gels. *Eur. J. Vasc. Endovasc. Surg.* 49(6):747, 2015. 10.1016/j.ejvs.2015.03.048.

[56] Velazquez, O. C., R. Snyder, Z. J. Liu, R. M. Fairman, and M. Herlyn. Fibroblast-dependent differentiation of human microvascular endothelial cells into capillary-like 3-dimensional networks. *FASEB J.* 16:1316–1318, 2002. 10.1096/fj.01-1011fje. 12060671

[57] Syedain, Z. H., M. Lahti, G. Hiremath, et al. Evaluation of an engineered vascular graft exhibiting somatic growth in lambs to model repair of absent pulmonary artery branch. *Commun. Med.* 4:201, 2024. 10.1038/s43856-024-00614-8. PMC11480500 39406880

[58] Syedain, Z. H., L. A. Meier, J. W. Bjork, A. Lee, and R. T. Tranquillo. Implantable arterial grafts from human fibroblasts and fibrin using a multi-graft pulsed flow-stretch bioreactor with noninvasive strength monitoring. *Biomaterials.* 32:714–722, 2011. 10.1016/j.biomaterials.2010.09.019. PMC3042747 20934214

[59] Zippusch, S., K. F. W. Besecke, F. Helms, M. Klingenberg, A. Lyons, P. Behrens, A. Haverich, M. Wilhelmi, N. Ehlert, and U. Böer. Chemically induced hypoxia by dimethyloxalylglycine (DMOG)-loaded nanoporous silica nanoparticles supports endothelial tube formation by sustained VEGF release from adipose tissue-derived stem cells. *Regen. Biomater.* 8:rbab039, 2021. 10.1093/rb/rbab039. PMC8363767 34408911

[60] Helms, F., S. Zippusch, T. Aper, S. Kalies, A. Heisterkamp, A. Haverich, U. Böer, and M. Wilhelmi. Mechanical stimulation induces *Vasa Vasorum* capillary alignment in a fibrin-based *Tunica Adventitia*. *Tissue Eng Part A.* 28:818–832, 2022. 10.1089/ten.TEA.2022.0042. 35611972

[61] Táborská, J., Z. Riedelová, E. Brynda, P. Májek, and T. Riedel. Endothelialization of an ePTFE vessel prosthesis modified with an antithrombogenic fibrin/heparin coating enriched with bound growth factors. *RSC Adv.* 11(11):5903–5913, 2021. 10.1039/d1ra00053e. PMC8694727 35423133

[62] Galbraith, C. G., R. Skalak, and S. Chien. Shear stress induces spatial reorganization of the endothelial cell cytoskeleton. *Cell Motil. Cytoskeleton.* 40:317–330, 1998. 10.1002/(SICI)1097-0169(1998)40:4<317::AID-CM1>3.0.CO;2-8. 9712262

[63] Chen, L., H. Qu, B. Liu, B. C. Chen, Z. Yang, D. Z. Shi, and Y. Zhang. Low or oscillatory shear stress and endothelial permeability in atherosclerosis. *Front Physiol.* 15:1432719, 2024. 10.3389/fphys.2024.1432719. PMC11417040 39314624

[64] Cunningham, K. S., and A. I. Gotlieb. The role of shear stress in the pathogenesis of atherosclerosis. *Lab Invest.* 85(1):9–23, 2005. 10.1038/labinvest.3700215. 15568038

[65] Gui, L., M. J. Boyle, Y. M. Kamin, A. H. Huang, B. C. Starcher, C. A. Miller, M. J. Vishnevetsky, and L. E. Niklason. Construction of tissue-engineered small-diameter vascular grafts in fibrin scaffolds in 30 days. *Tissue Eng Part A.* 20:1499–1507, 2014. 10.1089/ten.TEA.2013.0263. PMC4011429 24320793

[66] Swartz, D. D., J. A. Russell, and S. T. Andreadis. Engineering of fibrin-based functional and implantable small-diameter blood vessels. *Am. J. Physiol. Heart Circ. Physiol.* 288:H1451–H1460, 2005. 10.1152/ajpheart.00479.2004. 15486037

[67] Row, S., H. Peng, E. M. Schlaich, C. Koenigsknecht, S. T. Andreadis, and D. D. Swartz. Arterial grafts exhibiting unprecedented cellular infiltration and remodeling in vivo: the role of cells in the vascular wall. *Biomaterials.* 50:115–126, 2015. 10.1016/j.biomaterials.2015.01.045. PMC4350022 25736502

[68] Devillard, C. D., and C. A. Marquette. Vascular tissue engineering: challenges and requirements for an ideal large scale blood vessel. *Front. Bioeng. Biotechnol.*9:721843, 2021. 10.3389/fbioe.2021.721843. PMC8522984 34671597

[69] Mitchell, T. C., N. L. Feng, Y. T. Lam, P. L. Michael, M. Santos, and S. G. Wise. Engineering vascular bioreactor systems to closely mimic physiological forces *in vitro*. *Tissue Eng. Part B Rev.* 29:232–243, 2023. 10.1089/ten.TEB.2022.0158. 36274223

[70] García Sáez, D., B. Zych, A. Sabashnikov, C. T. Bowles, F. De Robertis, P. N. Mohite, A. F. Popov, O. Maunz, N. P. Patil, A. Weymann, T. Pitt, L. McBrearty, B. Pates, R. Hards, M. Amrani, T. Bahrami, N. R. Banner, and A. R. Simon. Evaluation of the organ care system in heart transplantation with an adverse donor/recipient profile. *Ann. Thorac. Surg.* 98:2099–2105, 2014. 10.1016/j.athoracsur.2014.06.098. 25443013

