# Supplementary material for: Generation of Fibrin-Based Aortic Vessels with Layer-Specific Cell Architecture Under Pulsatile Perfusion in a Clinical Organ Care System
Source: Ann Biomed Eng. 2026 Mar 27;54(11):3961–76. doi: 10.1007/s10439-026-04087-9 (PMC13694387; doi:10.1007/s10439-026-04087-9)
Supplement: Supplementary file 1 [file 10439_2026_4087_MOESM1_ESM.docx]

**Supplementary Material:**

**Supplementary Fig. 1**

Morphological evaluation (a, c) and immunostaining for calponin on chamber slides (b, d) of adipogenic-derived stem cells before (a, b), and after (c, d) pre-differentiation towards smooth muscle cells. Calponin was stained in red. Cell nuclei were counterstained using 4′,6-Diamidin-2-phenylindol (DAPI). Scale bar = 100 µm

**Supplementary Fig. 2**

SMCs were embedded in the *Media*-equivalent and stained for calcein and ethidium-homodimer after 7 days of incubation. A homogenous distribution and cell viability was observed (a). Cell viability was quantified in 10× magnification (b). Scale bares a = 500 µm, b = 100 µm

**Supplementary Fig. 3**

Cryosection of the *Intima*-, *Media*-, and *Adventitia-*equivalents. RFP-labeled HUVECs are visualized in red in the *Intima* and *Adventita*-equivalent. α-SMA-positive SMCs are visualized in green in the *Media*. Cell nuclei were stained with DAPI (blue). Scale bar = 500 µm

**Supplementary Fig. 4**

Assessment of the *Adventitia*: The outer surface of static (a, b) or dynamic (c, d) vascular grafts exhibited the formation of capillary tubes by RFP-HUVECs in the *Adventitia*-equivalent. Capillary formation was analyzed in 6 fields per vessel using the software AngioTool in 4× magnification. Scale bar (a, c) = 100 µm, (b, d) = 500 µm

**Supplementary Table 1**

| Time | Perfusion Time | Flow L/min | AOP Mean mmHg | AOP Systolic mmHg | AOP Diastolic  mmHg | Temp °C |
| --- | --- | --- | --- | --- | --- | --- |
| 14:52:16; 14:54:16; 14:56:16; 14:58:16; 15:00:16; 15:02:16; 15:04:16; 15:06:16; 15:08:16; 15:10:16; 15:12:16; 15:14:16; 15:16:16; 15:18:16; 15:20:16; 15:22:16; 15:24:16; 15:26:16; 15:28:16; 15:30:16; 15:32:16; 15:34:16; 15:36:16; 15:38:16; 15:40:16; 15:42:16; 15:44:16; 15:46:16; 15:48:16; 15:50:16; 15:52:16; 15:54:16; 15:56:16; 15:58:16; 16:00:16; 16:02:16 | 103:54:00; 103:56:00; 103:58:00; 104:00:00; 104:02:00; 104:04:00; 104:06:00; 104:08:00; 104:10:00; 104:12:00; 104:14:00; 104:16:00; 104:18:00; 104:20:00; 104:22:00; 104:24:00; 104:26:00; 104:28:00; 104:30:00; 104:32:00; 104:34:00; 104:36:00; 104:38:00; 104:40:00; 104:42:00; 104:44:00; 104:46:00; 104:48:00; 104:50:00; 104:52:00; 104:54:00; 104:56:00; 104:58:00; 105:00:00; 105:02:00; 105:04:00 | 01.50; 01.50; 01.49; 01.50; 01.50; 01.49; 01.50; 01.50; 01.50; 01.50; 01.50; 01.50; 01.49; 01.50; 01.50; 01.50; 01.50; 01.50; 01.50; 01.50; 01.50; 01.50; 01.50; 01.50; 01.50; 01.50; 01.50; 01.50; 01.50; 01.50; 01.50; 01.50; 01.50; 01.50; 01.50; 01.50 | 91; 91; 90; 91; 91; 90; 91; 91; 91; 91; 91; 91; 90; 91; 91; 91; 91; 91; 91; 91; 91; 91; 91; 91; 91; 91; 91; 91; 91; 91; 91; 91; 91; 91; 91; 91 | 124; 124; 124; 124; 124; 124; 125; 125; 125; 125; 125; 125; 125; 125; 125; 126; 125; 125; 125; 125; 125; 125; 125; 125; 125; 125; 125; 125; 125; 125; 125; 125; 125; 124; 124; 124 | 60; 60; 59; 60; 60; 60; 60; 60; 60; 60; 60; 60; 59; 60; 60; 60; 59; 59; 59; 60; 60; 60; 60; 59; 60; 60; 60; 60; 60; 60; 60; 60; 60; 60; 60; 60 | 36.9; 36.9; 36.9; 36.9; 36.9; 36.9; 36.9; 36.9; 36.9; 36.9; 36.9; 36.9; 36.9; 36.9; 36.9; 36.9; 36.9; 36.9; 36.9; 36.9; 36.9; 36.9; 36.9; 36.9; 36.9; 36.9; 36.9; 36.9; 36.9; 36.9; 36.9; 36.9; 36.9; 36.9; 36.9; 36.9 |
